# An ultra-low-cost electroporator with microneedle electrodes (ePatch) for SARS-CoV-2 vaccination

**DOI:** 10.1073/pnas.2110817118

**Published:** 2021-10-20

**Authors:** Dengning Xia, Rui Jin, Gaurav Byagathvalli, Huan Yu, Ling Ye, Chao-Yi Lu, M. Saad Bhamla, Chinglai Yang, Mark R. Prausnitz

**Affiliations:** ^a^School of Chemical and Biomolecular Engineering, Georgia Institute of Technology, Atlanta, GA 30332;; ^b^School of Pharmaceutical Sciences (Shenzhen), Sun Yat-sen University, Shenzhen 518107, China;; ^c^Department of Microbiology and Immunology, Emory University School of Medicine, Atlanta, GA 30322;; ^d^Emory Vaccine Center, Emory University School of Medicine, Atlanta, GA 30322;; ^e^School of Electrical and Computer Engineering, Georgia Institute of Technology, Atlanta, GA 30332;; ^f^Wallace H. Coulter Department of Biomedical Engineering at Emory University and Georgia Tech, Georgia Institute of Technology, Atlanta, GA 30332

**Keywords:** SARS-CoV-2, COVID-19 DNA vaccine, skin electroporation, piezoelectricity, microneedle array

## Abstract

Vaccination against severe acute respiratory syndrome coronavirus 2 (SARS-CoV-2) and other pathogens with pandemic potential requires safe, protective, inexpensive, and easily accessible vaccines that can be developed and manufactured rapidly at a large scale. DNA vaccines can achieve these criteria, but induction of strong immune responses has often required bulky, expensive electroporation devices. Here, we report an ultra-low-cost (<1 USD), handheld (<50 g) electroporation system utilizing a microneedle electrode array (“ePatch”) for DNA vaccination against SARS-CoV-2. The low cost and small size are achieved by combining a thumb-operated piezoelectric pulser derived from a common household stove lighter that emits microsecond, bipolar, oscillatory electric pulses and a microneedle electrode array that targets delivery of high electric field strength pulses to the skin’s epidermis. Antibody responses against SARS-CoV-2 induced by this electroporation system in mice were strong and enabled at least 10-fold dose sparing compared to conventional intramuscular or intradermal injection of the DNA vaccine. Vaccination was well tolerated with mild, transient effects on the skin. This ePatch system is easily portable, without any battery or other power source supply, offering an attractive, inexpensive approach for rapid and accessible DNA vaccination to combat COVID-19, as well as other epidemics.

## Significance

Low-cost and rapidly distributable vaccines are urgently needed to combat COVID-19 and future pandemics, especially for developing countries and other low-resource settings. DNA vaccines are inexpensive, rapidly developed, and safe, but require bulky and expensive electroporation devices for effective vaccination, which presents challenges to affordable and mass vaccination. We developed an ultra-low-cost (<1 USD), handheld (<50 g), battery-free electroporation system combining a thumb-actuated piezoelectric pulser and a microneedle electrode array skin interface for DNA vaccination against COVID-19, which was shown to be immunogenic and well-tolerated in animal studies. This study provides a proof-of-concept that DNA vaccination against epidemics can be achieved using an ultra-low-cost electroporator that is inexpensive enough for single use and robust enough for repeated use if desired.

Severe acute respiratory syndrome coronavirus 2 (SARS-CoV-2) is highly transmissible between humans and has created a global public health crisis resulting in over 4.3 million deaths globally, with the case counts still rapidly increasing and more contagious variants of the virus emerging (1). This pandemic presents an unprecedented global challenge to mitigate the further spread and rising death counts of COVID-19. A number of vaccines against COVID-19 have been introduced and are being made available in certain countries, with other countries having limited or no supplies (2). Access to messenger RNA (mRNA)-based vaccines has sometimes been limited by strict refrigeration requirements as low as −80 °C, and safety concerns have emerged around vaccines using a viral vector (3).

Synthetic DNA vaccines offer many of the advantages of mRNA vaccines, including rapid and low-cost development and manufacturing. Unlike mRNA vaccines, DNA vaccines are thermostable and can be cold-chain free, and also do not require the use of live virus. Indeed, at least 10 DNA vaccines for COVID-19 are in clinical trials globally, and at least 16 are in preclinical development (4). However, the historic challenge of DNA vaccines has been a concern with poor immunogenicity in larger animals and humans. Ongoing efforts to enhance immunogenicity focus on DNA platform optimization using techniques such as codon optimization, alternative delivery strategies such as electroporation and gene guns, and the use of adjuvants (5). Among these, electroporation has been notably successful, with 100- to 1,000-fold enhancements in plasmid delivery and gene expression relative to injection alone (6). In fact, DNA vaccination with skin electroporation has been shown to increase antigen-specific CD4^+^ and CD8^+^ T cell responses, IFN-γ levels, and humoral immune responses (7).

In recent studies, DNA vaccines delivered using electroporation were efficacious in phase II and III clinical trials (8), and 47 out of 70 clinical trials (from ClinicalTrials.gov, 2010–2017, excluding naked DNA injection) for plasmid DNA−based therapy have used electroporation (9). The most advanced DNA vaccine for COVID-19 currently in Phase II/III clinical trials also uses electroporation (10). Electroporation facilitates DNA vaccination by transiently breaking down cell membranes to drive DNA into cells, which can lead to the expression of the SARS-CoV-2 spike protein antigen. Plasma membrane poration requires the application of microsecond to millisecond pulses that generate electric fields of hundreds to thousands of volts per centimeter (11). The use of electroporators has, however, been greatly limited due to high equipment costs (thousands of US dollars), lack of portability (>5 kg), need for electricity, complex manufacturing, and difficult scaling up. These limitations reduce access by patients and clinics in low-resource settings, such as in developing countries. These limitations are particularly notable in pandemic scenarios, such as COVID-19, where traditional electroporators would be challenging to rapidly mass-produce and distribute. Thus, there is a need for an inexpensive, safe, effective, and easily accessible electroporation platform to administer DNA vaccines that can be rapidly scaled in response to outbreaks, such as COVID-19.

To address this need for effective DNA vaccine delivery strategies to curb the COVID-19 pandemic and future ones, we developed an ultra-low-cost, portable, and easy-to-use microneedle electrode array (MEA) electroporator for enhancing the immunogenicity of a SARS-CoV-2 DNA vaccine. This electroporation system consists of a piezoelectric pulse generator and a metal MEA, which, together, we call the ePatch. The pulse generator is derived from a common household piezoelectric stove lighter, which is currently mass-produced by the billions. In this way, our pulse generator is easily accessible and costs as little as $0.23 (US dollars) to manufacture (12). In our prior study, we have demonstrated, in vitro, that this pulse generator was able to transform electrocompetent *Escherichia coli* with a transformation efficiency comparable to a conventional benchtop electroporator that was >10,000 times more costly and >100-fold bigger (12). Moreover, the piezoelectric pulse generator produces bipolar, oscillatory pulses, which can electroporate cells more effectively compared to conventional exponential decay or square-wave pulses (13).

Because the piezoelectric pulses are of microsecond duration, effective electroporation benefits from a field strength of >1,000 V/cm (14). To achieve such a high field strength, we used MEAs with very close (i.e., 0.9 mm) spacing, such that piezoelectric pulses of hundreds of volts can be used to achieve the very large, required field strengths. Much larger voltages would be needed to achieve this field strength if we used conventional clamp electrodes or penetrating electrodes with spacings of many millimeters. A second benefit of using MEAs is that they can be used to target delivery to the skin, which has been shown to provide greater immunogenicity for DNA and other vaccines compared to vaccination in the muscle (15, 16). Finally, the microneedles are just 650 μm long, which can concentrate the electric field in the epidermis, which is especially rich in antigen-presenting cells, and keep electric fields away from stimulating sensory and motor nerves deeper in the dermis or muscle tissue below. Microneedles are an inexpensive and simple-to-use technology that has previously been employed for vaccine delivery to the skin (without electroporation) in preclinical and clinical studies (15, 17). Alternatively, prior studies in our laboratory have demonstrated microneedles functionalized as electrodes for delivery of electric pulses to cause electroporation in cells in vitro (18, 19).

In this study, we tested the ePatch using a DNA vaccine that expresses the SARS-CoV-2 spike protein, which is the target antigen for most COVID-19 vaccines under development (20). Here, we present the device design, characterize its performance in vitro, and study its effects in vivo including gene expression in the skin, immune responses of a SARS-CoV-2 DNA vaccine, virus neutralization, and tolerability evaluation to assess the enhanced immunogenicity and safety profile of this ultra-low-cost electroporation system with MEA electrodes (ePatch).

## Results

### Design of the ePatch.

**Fig. 1.**
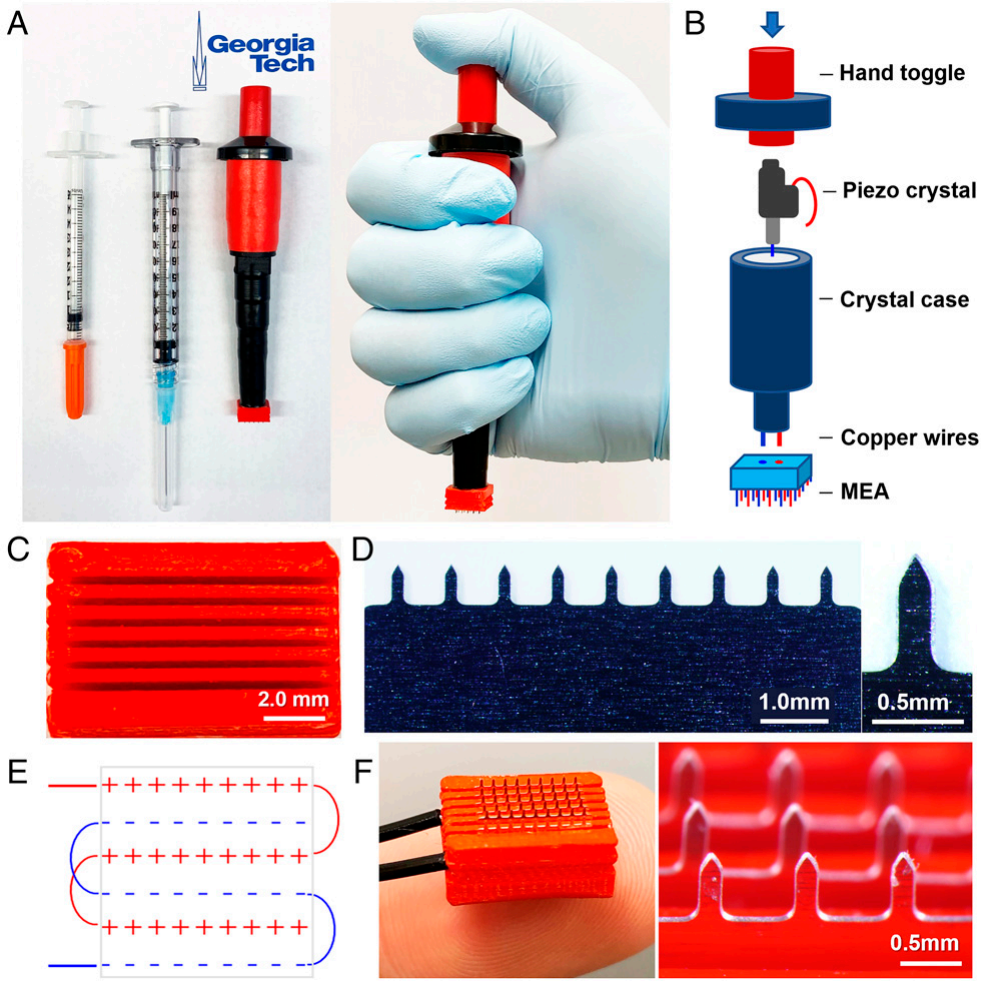
Design of electroporator with piezoelectric pulse generator and MEA. (*A*) The ePatch is shown with 0.5- and 1-mL syringes for comparison (*Left*) and shown being held in position before activation (*Right*). (*B*) Components of the ePatch. (*C*) The 3D printed insulating holder of microneedle electrodes in MEA to accurately position and electrically isolate microneedle electrodes of opposite polarity. (*D*) A row of stainless steel microneedle electrodes (*Left*) and a single microneedle (*Right*). (*E*) Diagram showing the configuration of the electrodes in an MEA. (*F*) Assembled MEA (*Left*) and magnified view of a section of the MEA in an assembled ePatch (*Right*).

The design criteria for the ePatch were to administer electric pulses suitable for electroporation of cells in the skin’s epidermal layer using a simple and low-cost device that can be quickly mass-produced. The resulting design consists of a piezoelectric pulse generator and a metal MEA (Fig. 1). The electric pulses are generated based on piezoelectricity, a technique derived from the mechanism found in a common household gas lighter. The pulses are generated using a spring-latch mechanism wherein a hammer strikes a piezoelectric crystal, producing a powerful mechanical force converted into high-voltage electrical energy that is used to generate a spark when applied across an air gap (i.e., when operated as a lighter), but can be used to pass current through tissue using microneedle electrodes. We previously described the theoretical principles of this spring-latch mechanism and its advantages in enabling tunable and consistent electric pulses independent of user force (12).

The MEA was fabricated by assembling six rows of stainless steel microneedles measuring 650 μm in length and 200 μm by 50 μm in cross-section that tapers to a sharp tip mounted in a 3D-printed insulating holder made of polylactic acid (Fig. 1*C*). Each row of electrodes with the same electrical polarity consists of nine microneedles each separated by 0.8-mm spacing within each row, and with rows separated by 0.9-mm spacing (Fig. 1 *D* and *E*). This close spacing serves to enable the large electric field strength needed for electroporation using microsecond pulses. The piezoelectric pulse generator is connected to the MEA using wire for positive and negative terminals (Fig. 1*F*). In use, the MEA is pressed against the skin so that the microneedles penetrate across the skin’s stratum corneum barrier to enter the viable epidermis and superficial dermis, after which the user presses the thumb toggle to administer the pulses.

### Analysis of High-Voltage Pulses and the Electric Field Generated by the ePatch.

**Fig. 2.**
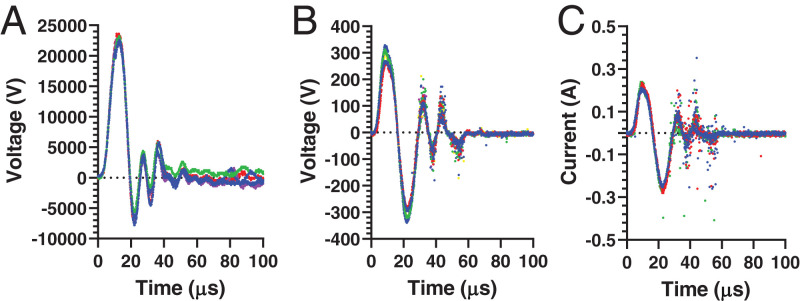
Representative electrical output profiles for piezoelectric pulser used for electroporation. Piezoelectric pulser activated by open-circuit measurements made (*A*) by connecting the pulser leads to the oscilloscope (voltage profile) and (*B* and *C*) by pulsing in porcine skin ex vivo (*B*, voltage profile; *C*, current profile). Multiple replicate voltage and current profiles are shown (*n* = 4 to 6).

Using a high-voltage probe and oscilloscope, we first determined the voltage outputs from directly connecting the wire of the piezo pulse generator to an oscilloscope. The outputs generated pulses with peak positive voltages and peak negative voltages of 22.7 ± 0.3 kV and −6.8 ± 0.8 kV, respectively (Fig. 2*A*). The peak positive voltage was achieved after 10.3 ± 0.7 μs, and the oscillating voltage output that followed decayed within ∼100 μs (Fig. 2*A*).

When applied to porcine skin ex vivo using an MEA as electrodes, we found that the peak positive and negative voltage outputs were 296 ± 25 V and −313 ± 20 V, respectively (Fig. 2*B*). The time to peak voltage was 8.6 ± 0.3 μs. Here, the voltage was much lower due to the lower impedance of skin compared to open-circuit measurement. The electric pulses were in the form of a bipolar oscillating decaying waveform, which is characteristic of piezoelectric pulses (21).

For a comparative analysis, we also generated electric pulses using a conventional bench electroporator commonly employed for laboratory transfections (22) and coupled to an MEA in porcine skin ex vivo. This pulser generated monopolar exponential decay pulses of 32.1 ± 0.2 V or 99 ± 5 V with 52.2 ± 4.4 ms or 50.1 ± 2.7 ms pulse durations (i.e., exponential decay time constant), respectively (*SI Appendix*, Fig. S1*C*). These millisecond, monopolar pulses are more typical of those used for conventional electroporation (23, 24), which contrast with the microsecond, oscillatory pulses generated by the ePatch.

We also measured the electric current through the skin during pulses administered using ePatch, which showed an oscillating decaying waveform that was similar in shape to the voltage waveform and achieved a peak current of 0.27 ± 0.01 Å (Fig. 2*C*). For the conventional benchtop electroporator, the peak currents through the skin were 0.015 ± 0.001 Å and 0.253 ± 0.002 Å when the 32- and 99-V pulses, respectively, were applied (*SI Appendix*, Fig. S1*D*). The apparent electrical impedance of skin (i.e., characterized as peak voltage divided by peak current) was 1,160 Ω during ePatch pulsing and 2,130 Ω or 390 Ω during pulsing by the conventional electroporator (at 32 V or 99 V, respectively).

**Fig. 3.**
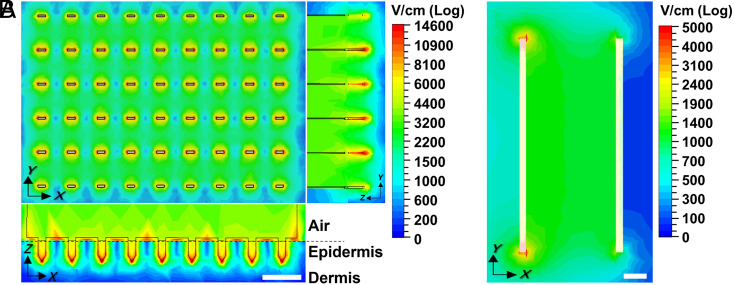
Electric field strength distribution in the skin determined by computer simulation. Peak electric field strength is shown when applying a 300-V pulse like those from the ePatch using an (*A*) MEA or (*B*) clamp electrode. Field strength distribution is shown in *A* from above the MEA (*Upper Left*) and as side views (*Bottom Left* and *Right*), and in *B* from above the skin. Dermal−epidermal junction is indicated by the dashed line. (Scale bar: 1 mm.)

To better understand the electric field distribution in the skin when applying pulses using an MEA, we modeled the electric field strength in the skin during electroporation (Fig. 3*A*). For the MEA, the electric field strength was highest surrounding each needle electrode, especially near the tip, where electrode curvature is known to increase electric field strength (25). The electric field strength was weakest between electrodes of the same polarity. The electric field also did not penetrate deeply into the tissue below the electrodes, dropping off on a length scale of hundreds of microns. In this way, the electric field was localized to the epidermis and upper layer of the dermis, which contain abundant antigen-presenting cells, such as epidermal Langerhans cells and dermal dendritic cells, and have efficient drainage to lymph nodes, all of which can enhance vaccine immunogenicity.

The threshold value for reversible electroporation depends on the duration of exposure to the electric field (11). For the millisecond-long pulses, the electroporation threshold is expected to be on the order of 400 V/cm to 600 V/cm (11, 26), while, for the microsecond pulse duration (as in the ePatch), the threshold is increased to 1.0 kV/cm to 1.5 kV/cm (27–29). When simulating 300-V pulses like in the ePatch, the highest electric field strength in the tissue is 15 kV/cm immediately next to the electrodes, but most of the tissue experiences field strengths of 2 kV/cm to 3 kV/cm (Fig. 3*A*), which is higher than the threshold necessary for successful electroporation, but still low enough to avoid extensive cell killing (30). In this way, we might expect highly localized cell death adjacent to the electrodes (red regions in Fig. 3*A*) as well as small regions that are not electroporated between electrodes of the same polarity (blue regions in Fig. 3*A*), but most tissue experiences a field strength expected to cause reversible electroporation (green regions in Fig. 3*A*).

We further compared these simulations to the field strength in the skin generated using a conventional clamp electrode at the same voltage (300 V), and found that the large spacing (i.e., 3.9 mm) of the clamp electrode produced much weaker electric field strengths compared to the MEA (Fig. 3*B*). The field strength only exceeded 1 kV/cm in a portion of the space between the electrodes (green regions in Fig. 3*B*), and only went above 1.5 kV/cm at the very edges of the electrode (red regions in Fig. 3*B*). Penetrating needle electrodes that are also used in current electroporation protocols would suffer from the same limitations as the clamp electrodes due to their similarly large spacing between electrodes.

We finally investigated field strength in the skin during the application of representative pulses from a conventional electroporator (30 and 100 V) using a clamp electrode or MEA (*SI Appendix*, Fig. S3). The 30-V pulses with clamp electrode produced very low field strengths mostly below 300 V/cm, which does not achieve the expected electroporation threshold for millisecond pulses. Application of 30-V pulses with the closely spaced MEA enabled tissue immediately adjacent to the electrodes to reach 400 V/cm to 600 V/cm, but most of the tissue experienced much weaker electric fields. When using 100-V pulses, the clamp electrode achieved field strength expected to electroporate in some of the tissue, and the MEA produced electric fields strong enough for electroporation in most of the tissue.

### Robust Reporter Gene Transfection by ePatch.

To evaluate the effects of the ePatch on plasmid delivery and transfection, we delivered a green fluorescent protein (GFP)-encoding DNA plasmid to rat skin in vivo. The level of gene expression was measured by in vivo imaging of GFP fluorescence over time.

**Fig. 4.**
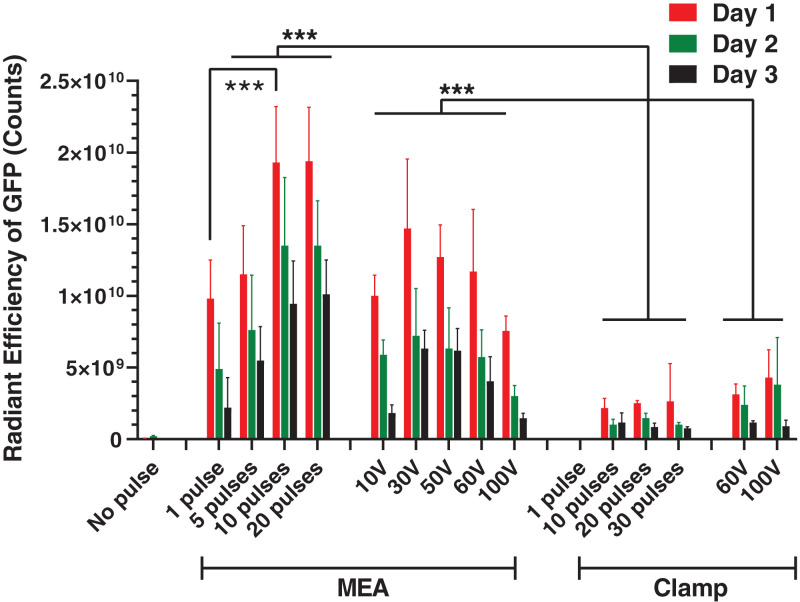
GFP expression in rat skin after electroporation. Radiant efficiency of GFP fluorescence in the skin on different days after delivery of GFP reporter plasmid by electroporation using an ePatch giving 1 to 20 pulses of ∼300 V with a waveform like that shown in Fig. 2*B* or using a conventional exponential decay electroporation pulser at controlled peak voltage (10 V to 100 V) with decay time constants (τ = 49 ms to 57 ms). Pulses were applied using an MEA or a clamp electrode. Data represent mean ± SD (*n* = 5 or 6) (***P < 0.001).

We first tested the effect of high field strength, microsecond pulses using the ePatch, and found that a single pulse was able to generate visible GFP expression (Fig. 4). More pulses increased GFP expression up to 10 pulses (*P* = 0.001); increasing to 20 pulses did not increase GFP expression further (*P* > 0.05). For 3 d after electroporation, GFP expression decreased over time (*P* = 0.002). After 5 d, GFP fluorescence was undetectable, likely due to GFP protein degradation in the skin (31). The degree of GFP expression was relatively consistent, with relative SD values of 20 to 30%. Prior work has shown that the interindividual variability of gene expression and resulting titers within a group can be reduced by electroporation treatment (32–34).

As a negative control, we performed an intradermal (ID) injection of the GFP plasmid into the skin without electroporation, which resulted in barely detectable GFP transfection (Fig. 4). The ePatch increased GFP expression 416-fold relative to ID injection alone (*P* < 0.001).

**Fig. 5.**
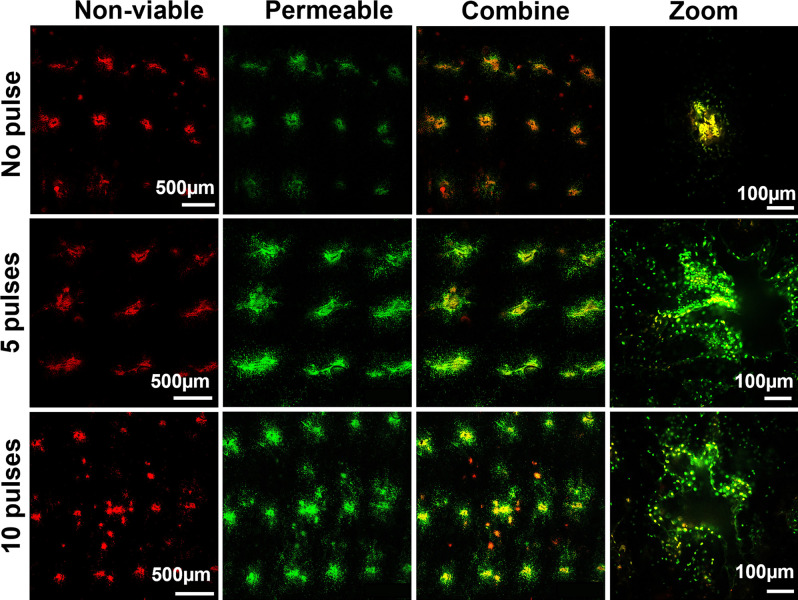
Cell membrane permeabilization and cell viability in mouse skin in vivo after electroporation by ePatch. Representative images show nonviable cells (red color) and cells with permeabilized membrane (green color) in the skin after electroporation with 0, 5, or 10 pulses by ePatch. Nonviable cells were identified in the mouse skin after insertion of MEA without electroporation (no pulse), suggesting that a small number of cells were damaged by microneedle electrode insertion alone. The red and green signals are colocalized in the insertion holes because nonviable cells are also permeable to the SYTOX Green. After 5 and 10 pulses, the red signal did not increase in the skin, while the green signal became more dispersed in the skin, suggesting transient cell permeability induced by the piezoelectric pulses with little effect on cell viability.

To better interpret these results, we carried out an additional experiment with a cell-impermeable green marker compound (SYTOX Green) present during electroporation to identify permeabilized cells and a red viability stain added afterward to identify nonviable cells. Inspection of the skin by microscopy showed that there was loss of cell viability at the sites of microneedle puncture into the skin, independent of electroporation, as indicated by the presence of red fluorescent cells (Fig. 5). This was probably due to damage from mechanical puncture by the microneedles. Application of 5 or 10 electroporation pulses from the ePatch did not appear to increase cell viability loss, but did cause increased cell permeabilization with the uptake of the green marker compound into viable cells surrounding the nonviable core at the site of each microneedle penetration. Microscopic examination of the skin surface showed only faint and transient evidence of skin damage at the sites of each microneedle electrode penetration, as discussed below.

We next tested the effect of moderate field strength, millisecond pulses using the MEA coupled with the conventional electroporator. Electroporation under these conditions yielded GFP expression that peaked at 30 V (*P* = 0.02) (Fig. 4). The peak GFP expression at 30 V was not significantly different from the peak value generated by the ePatch with 10 pulses (*P* = 0.055), whereas GFP expression at other voltages was significantly lower (*P* < 0.05). Similar to the ePatch, cells transfected by electroporation using millisecond pulses also had decay in GFP fluorescence for 3 d after electroporation (*P* = 0.008).

The dependence of GFP expression on voltage can be explained by a lesser degree of electroporation at 10 V versus 30 V, resulting in less transfection. Above 30 V, possible increased DNA delivery into cells was likely offset by the increased loss of cell viability caused by irreversible electroporation and tissue heating during the millisecond-long exposure to high electric field strengths. This interpretation is supported by additional skin imaging after the application of the red viability stain (*SI Appendix*, Fig. S4). Increased loss of cell viability is seen with increasing voltage, and tissue heating at the sites of microneedle electrode placement increased as well, reaching peak values up to 50 °C (*SI Appendix*, Fig. S4). Microscopic examination of the skin surface showed discoloration at the sites of each microneedle electrode penetration that persisted for at least 2 d, consistent with the observation of extensive cell death at the higher voltages using millisecond pulses (*SI Appendix*, Fig. S5). These findings are consistent with prior reports of apoptotic and necrotic death in the epidermis adjacent to invasive needle electrodes when using millisecond-long pulses (35).

As an additional comparison that addresses current methods of skin electroporation, we employed clamp electrodes (3.9-mm spacing) instead of MEAs. When pulsing with the microsecond piezoelectric pulse generator, a single pulse did not result in detectable GFP expression, but applying 10, 20, or 30 pulses produced GFP expression that was independent of the number of pulses (*P* > 0.05) (Fig. 4). Using the clamp electrode with millisecond pulses from the conventional electroporator, detectable GFP expression was found at 60 V, and was slightly increased when the voltage increased to 100 V (*P* > 0.05). The GFP expression was significantly lower with the clamp electrode than when using the MEA with either ePatch or conventional electroporator (*P* < 0.001).

Altogether, these results demonstrate that 1) using an MEA with close microneedle electrode spacing leads to high electric field strengths that make the microsecond pulses from the piezoelectric pulser effective for gene transfection, and even improves transfection performance of a conventional millisecond electroporator; 2) the microsecond pulsing minimizes tissue heating that appears to damage tissue when using millisecond pulses; and 3) high levels of DNA transfection and expression can be achieved by the ePatch.

### Gene Transfection in the Epidermis.

**Fig. 6.**
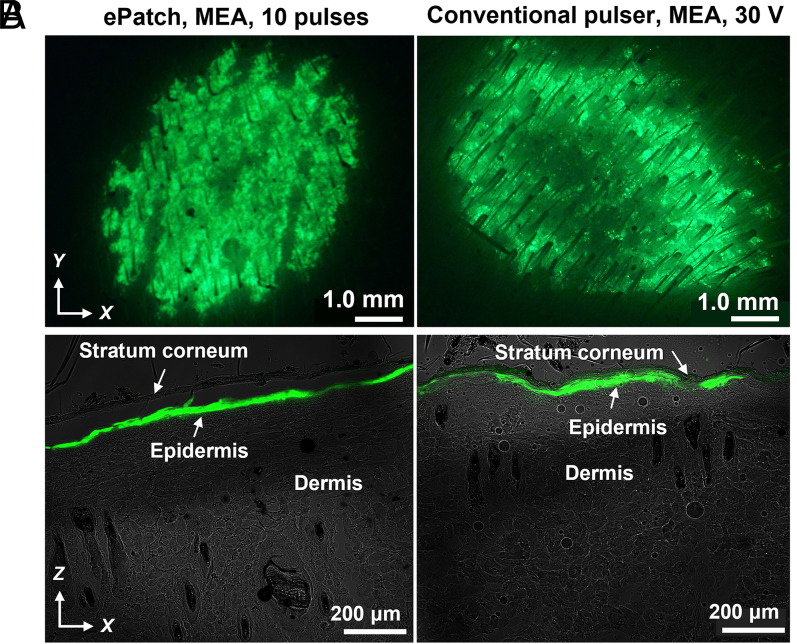
Fluorescence micrographs of rat skin imaged 1 d after delivery of GFP reporter plasmid by electroporation. Representative images are shown after electroporation using an MEA with 10 piezoelectric microsecond pulses administered by ePatch (*Left*) and with a single, exponential decay millisecond-long pulse (30 V, τ = 54 ms) administered by a conventional electroporation pulser (*Right*). After electroporation in vivo, skin was biopsied and imaged by (*A*) stereo fluorescence microscope on the skin surface and (*B*) laser scanning confocal microscopy as cryosections of the skin. The green color indicates GFP fluorescence. Skin anatomy is indicated in *B*.

To assess targeting of gene transfection to the epidermis, we performed histological analysis 1 d after DNA delivery. Electroporation with MEA using either microsecond pulses from the ePatch or using millisecond pulses from the conventional electroporator resulted in strong green fluorescence evident across the skin surface exposed to the MEA when viewed *en face* on the skin surface (Fig. 6*A*), and throughout the viable epidermis, with little evidence of GFP expression in dermis or stratum corneum, when viewed as a frozen histological section (Fig. 6*B*). These images show that the transfected cells were almost exclusively identified within the epidermal layer beneath the stratum corneum. In contrast, when electroporation was carried out using the clamp electrode, GFP fluorescence was less intense (consistent with the quantitative findings in Fig. 4). Moreover, although the transfected cells were mostly in the epidermis, we can also see evidence of GFP transfection in the deep dermal layer, notably in the hair follicles (*SI Appendix*, Fig. S6). These findings confirm the ability of the MEA to localize electroporation to the epidermis.

### Robust Immune Response and Viral Neutralization After SARS-CoV-2 DNA Vaccination in Mice.

After confirming that the ePatch can significantly augment gene expression in vivo, we evaluated the immunogenicity of a SARS-CoV-2 DNA vaccine delivered by ID injection with ePatch electroporation, ID injection without electroporation, and intramuscular (IM) injection at two different doses (10 and 100 μg of DNA) without electroporation.

**Fig. 7.**
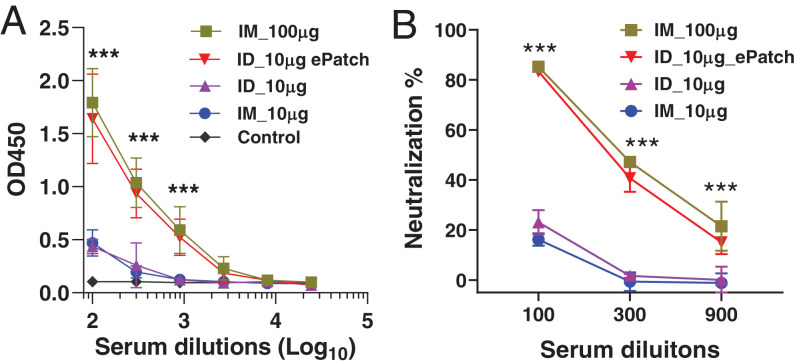
Humoral immune response and viral neutralization after SARS-CoV-2 DNA vaccination in mice. The mice were immunized at week 0 and week 4; blood samples were withdrawn at week 7. (*A*) IgG titer against SARS-CoV-2 spike surface protein in the mouse serum was expressed as absorbance at 450 nm (OD450) at different dilutions. (*B*) Neutralization of IgG against pseudovirus was analyzed at different dilutions of serum and expressed as neutralization percent for each dilution. Control: mice immunized by PBS; IM_10 μg and IM_100 μg: mice immunized with 10 and 100 μg DNA vaccine, respectively, of DNA vaccine by IM injection; ID_10 μg: mice immunized with 10 μg of DNA vaccine by ID injection; ID_10 μg_ePatch: mice immunized with 10 μg of DNA vaccine by ID injection followed by electroporation using 20 pulses by ePatch; *n* = 5 mice per group (***P < 0.001).

IM vaccination produced humoral immune responses that were higher at the 100-μg dose than the 10-μg dose, as measured by antigen-specific IgG titers (*P* < 0.001) and virus neutralization assay (*P* < 0.001) (Fig. 7). ID injection of 10 μg of DNA vaccine yielded results similar to IM vaccination at the same dose (*P* > 0.05). When ID vaccination was carried out with electroporation by ePatch, immune responses were significantly higher than for ID or IM vaccination without electroporation at the same DNA dose (*P* < 0.001). Moreover, ePatch vaccination with 10 μg of DNA was not significantly different from IM vaccination with 100 μg of DNA, and reached an endpoint titer at about 1:2,700 serum dilution (*P* > 0.5). In comparison, serum antibody responses induced by ID or IM injection of 10 μg of DNA reached an endpoint titer at about 1:300 serum dilution. These results indicate that the level of binding antibodies to the SARS-CoV-2 spike protein in the ePatch group was almost 10-fold higher than the level in the ID or IM injection groups, with a 10-μg DNA dose. This indicates at least a 10-fold dose sparing enabled by ePatch vaccination.

Finally, it is worth noting that with a 100-fold dilution of serum, 90% neutralization of antibody against SARS-CoV-2 pseudovirus was found for the low-dose ePatch and the high-dose IM injection groups, while only 20% neutralization was found for the low-dose IM and ID injection groups without electroporation (Fig. 7*B*). Altogether, this study demonstrates that the ePatch significantly improved immune responses to SARS-CoV-2 relative to IM or ID injection alone.

**Fig. 8.**
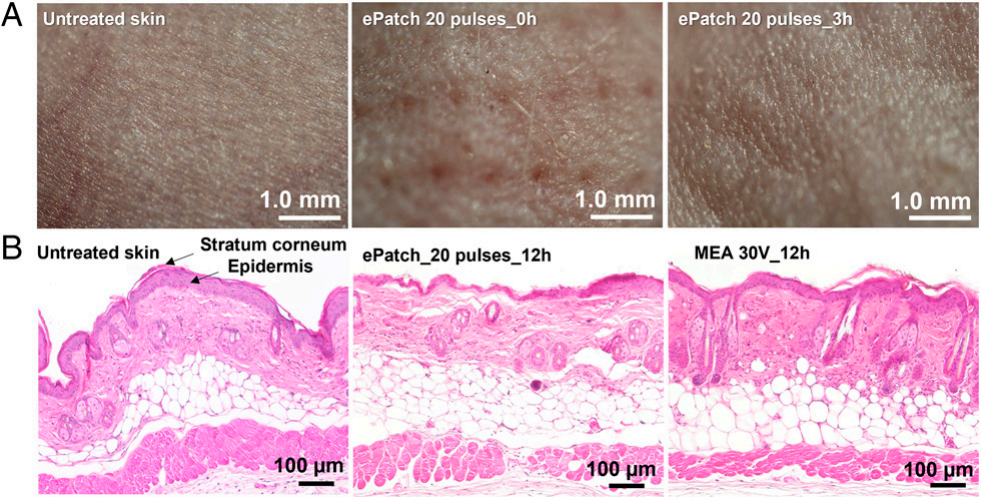
Histological examination of skin after electroporation in vivo. (*A*) Representative images obtained by stereo microscope are shown for untreated mouse skin and skin 0 h and 3 h after electroporation using 20 pulses by the ePatch in vivo. (*B*) Representative images obtained by brightfield microscopy of untreated mouse skin and skin electroporated with pulses by MEA combined with conventional millisecond electroporator (30 V, 55 ms) or ePatch with 20 pulses of microsecond duration. The skin was harvested 12 h after electroporation and H&E stained for examination.

Clinical and histological examination suggests that vaccination using the ePatch was very well tolerated. Imaging of the skin surface immediately after electroporation showed evidence of microneedle puncture and/or localized electroporation when viewed with magnification (Fig. 8*A*), Subsequent imaging after 3 h exhibited no residual evidence of the vaccination procedure. Histological examination of skin 12 h after ePatch vaccination showed no inflammatory markers (Fig. 8*B*). In contrast, high-voltage (100 V) millisecond pulsing caused extensive infiltration of inflammatory cells seen in the skin 12 h after electroporation (*SI Appendix*, Fig. S7). Clinical examination of the animals over the weeks that followed vaccination produced no significant findings. These data suggest that ePatch vaccination caused only mild, transient effects to skin that do not raise safety signals.

## Discussion

This study introduces a DNA vaccination method that benefits from the combination of two innovations: a piezoelectric-based power source for electroporation and an MEA that generates large electric fields targeted to the epidermis. This combination, in the form of the ePatch, was shown to enable DNA vaccination using a simple, ultra-low-cost system that can expand the reach and speed of vaccination against COVID-19 and future pandemics.

### Enables DNA Vaccination.

DNA vaccines have great promise as a low-cost, rapidly developed, and broadly applicable vaccination method well suited for pandemics like COVID-19, as well as routine use. DNA vaccines do not have the stability problems of mRNA vaccines requiring frozen storage and do not have the slow development and manufacturing timelines of many conventional vaccines. However, in order to make DNA vaccines effective in humans, methods to enhance their immune response are needed, like electroporation, as seen, for example, in the SARS-CoV-2 DNA vaccine developed by INOVIO Pharmaceuticals, which has achieved improved immune responses by using electroporation in phase 2/3 clinical trials (36). However, electroporation in this and other DNA vaccination studies requires an expensive electroporation device with a complex design powered by batteries or an electrical outlet and uses large needle electrodes that penetrate skin or muscle, which, altogether, limits rapid and widespread access to the vaccine.

The ePatch overcomes this significant barrier to practical application of DNA vaccination. The delivery system is very inexpensive, made of components that are already manufactured at scale, simple to operate by minimally trained health workers, powered without battery or electrical outlet, and minimally invasive. As proof of principle, this approach provided enhanced immune responses to SARS-CoV-2 DNA vaccination, demonstrating stronger humoral immune responses and viral neutralization compared to IM or ID vaccination without electroporation, and also exhibited good tolerability and no apparent safety concerns.

### Low Cost, Portable, and Rapidly Manufactured.

The ePatch was designed to enable rapid and widespread access to DNA vaccination, which is critical to combat COVID-19 and other pandemics, as well as to facilitate vaccination in hard-to-reach populations. We achieved this low cost by using a piezoelectric pulse generator found in disposable household gas lighters that are currently mass-produced (in billions) for pennies each (37, 38), and an MEA produced by lithographic etching technology and 3D printing in widespread use to make components for disposable consumer products that likewise cost just pennies each (39). The resulting cost, expected to be <1 USD, is several orders of magnitude lower than currently available electroporators that usually cost thousands of US dollars. While the ePatch is inexpensive enough to be completely disposed of after a single use, the piezoelectric pulser can, alternatively, be reused (as done when used as a lighter), with the MEA replaced after each use, for safety.

The ePatch is easily portable. It has handheld operation, weighs under 50 g, has a size less than 20 cm^3^, and requires no battery or power sources, which makes the ePatch simple to transport and operate by minimally trained personnel, especially in resource-limited and remote parts of the world. This contrasts with traditional electroporators, which are big, heavy, and complex to operate and require access to electricity, although electroporation devices are being developed for clinical use to overcome some of these limitations (*SI Appendix*, Fig. S8).

### Electric Field Localized to the Epidermis.

The MEA used in this study has an array of 54 microneedle electrodes measuring 650 μm long with 0.9-mm spacing that localize the electric field to the epidermis. Other electroporators use much longer, fewer, and more widely spaced penetrating needle electrodes or clamp electrodes that distribute the electric field throughout the skin and into the hypodermis (40, 41).

It is important to target the electric field to the epidermis for improved immunogenicity and reduced side effects. Unlike the dermis, which is largely acellular, the epidermis is densely populated with cells, including keratinocytes as well as potent antigen-presenting cells, such as dendritic cells, including Langerhans cells (42, 43). Targeting antigen to these epidermal cells has been shown to improve immune responses compared to IM injection (44, 45). While electroporation of the dermis has less value, diffusion of antigens produced in epidermal cells into the upper dermis is beneficial, due to the presence of dermal dendritic cells and a rich vasculature that enables drainage to lymph nodes, which also increases immunogenicity (46).

Localizing the electric field to the epidermis can reduce nerve stimulation, thereby making electroporation more tolerable. Of particular concern is stimulation of motor nerves and muscle cells below the skin, which can cause violent twitching reported for skin electroporation in other contexts (47, 48). Indeed, we saw this in our animal studies, where electroporation with the clamp electrode caused strong muscle contractions at the site of electroporation upon application of each pulse. The animals were anesthetized, which indicates a direct stimulation of action potentials in muscle cells and/or motor nerves. These contractions were not seen when electroporating with MEAs that localized the electric field superficially, far away from muscles.

### Dense Electrode Spacing.

Electroporation requires strong electric fields on the order of 10^3^ V/cm, with shorter pulses requiring larger field strengths (49, 50). This means that more closely spaced electrodes can electroporate with lower voltages. By using MEAs with 0.9-mm spacing, we were able to electroporate skin with microsecond pulses from the ePatch with 300-V output, and achieved electroporation using millisecond pulses of just 10 V to 30 V from a conventional electroporator. These voltages are much lower than the 50 V to 200 V usually used with conventional millisecond pulsers (47, 49), which reduces device cost and complexity, and increases safety. Close electrode spacing also decreases electric field penetration depth into the skin (51), which facilitates epidermal targeting and reduces nerve stimulation.

### Narrow Bipolar Oscillating Pulse Profile.

We used a bipolar oscillating pulse for electroporation instead of a monopolar exponential decay or square-wave pulse that is conventionally used for electroporation (24, 50, 52). This bipolar oscillating pulse is a natural result of the compression and extension of piezoelectric crystals induced by a spring shock in the case containing the piezoelectric crystal. Compared to conventional monopolar pulses, multiple studies showed that bipolar oscillating microsecond pulses produce not only a dielectric breakdown of the cell membrane but also a sonicating motion in the cell membrane, inducing more effective cell poration (13, 53). Furthermore, oscillating pulses can provide better cell viability by avoiding polarizing the cell membrane beyond the critical potential for an extensive period, therefore preventing irreversible rupture of the cell membrane (53, 54).

### Comparison to Prior Studies.

We can compare findings from this study to prior reports in the literature on DNA vaccination enhancement by electroporation. In terms of gene expression, our study found a >400-fold increase in GFP expression in rat skin using the ePatch. Prior studies in rodents have similarly reported on the order of 100-fold increases in gene expression in various tissues of the body (32, 50, 55–58). Considering dose sparing, we found at least 10-fold dose sparing using the ePatch. Studies in the literature, for example by Genetronics (now INOVIO Pharmaceuticals) and Ichor Medical Systems, similarly report fivefold to 10-fold dose sparing (59, 60). Measuring immune response to DNA vaccination, our study saw an almost 10-fold increase in binding antibody titer with the ePatch compared to IM or ID injection at the same dose (10 μg). Other studies in rodents (including INO-4800, which is a SARS-CoV-2 vaccine currently in phase 3 clinical evaluation by INOVIO) reported two fold to 40-fold increased titers (61–63).

### Limitations and Expectations.

This study has several limitations. While DNA delivery, cell transfection, and antigen-specific immune responses were demonstrated, the study was conducted using a small-animal model with relatively small group size. Future studies should include larger animal species with larger numbers of animals and ultimately progress to human clinical trials. In addition, immune responses were characterized only in terms of antigen-specific antibody titers and pseudovirus neutralization. A more detailed immunological characterization is needed, including live virus challenge studies to evaluate protection against SARS-CoV-2 and other pathogens. Finally, safety and skin tolerability need additional study.

The devices in this study were hand-assembled prototypes. Additional work will be needed to develop an integrated device for low-cost mass production. Further optimization of electric pulse parameters and other aspects of ePatch operation will also be needed to optimize immunogenicity and safety in preclinical and clinical studies. Our current protocol involves ID vaccine injection followed by pulse application. Future studies should develop a single-step process, for example, by coating DNA vaccines on the microneedles for localized dissolution in the skin (64).

## Conclusions

DNA vaccination requires improved delivery for robust immunity in humans. Electroporation is an effective way to increase DNA transfection and immune response, but currently requires bulky, costly and complex instrumentation, which limits access, especially in a pandemic. To address this problem, we developed a low-cost, portable, and rapidly deployable electroporation system powered by an ultra-low-cost piezoelectric household stove lighter element that emits bipolar oscillating electric pulses well suited for electroporation. This ePatch administers the pulses using an MEA that has dense electrode spacing to create high field strength from moderate-voltage pulses and has a short microneedle length to target the electric field to the epidermis.

We demonstrated, in rats and mice, that the ePatch selectively transfected cells in the epidermis using microsecond pulses with no evidence of lasting damage to the skin. In contrast, electroporation using millisecond pulses from a large, costly, conventional electroporation device exhibited significant damage at the site of each microneedle electrode penetration in the skin. When used to administer a DNA vaccine for the SARS-CoV-2 spike protein, the ePatch produced robust humoral immune responses and viral neutralization, demonstrating at least 10-fold dose sparing compared to ID or IM injection without electroporation. We conclude that microsecond, oscillating pulses from an ultra-low-cost piezoelectric power source and administered using a densely spaced MEA with submillimeter microneedle electrodes can be used for DNA vaccination against SARS-CoV-2 and potentially other pathogens and can expedite development, reduce cost, and increase access to life-saving vaccines.

## Materials and Methods

### Animal and Plasmid.

All animal experiments were performed in compliance with the Institutional Animal Care and Use Committee guidelines of Emory University and the Georgia Institute of Technology. Adult female Wistar rats (250 g to 300 g) and 6- to 8-wk-old female BALB/c mice were supplied by Charles River Laboratories. The animals were kept in a 12 h/12 h light/dark cycle at the animal care facility, given free access to diet and water, and acclimatized for at least 7 d before the experiments. SYTOX Green was obtained from Thermo Fisher Scientific, ethidium bromide (EB) was obtained from Sigma-Aldrich, and poly(lactic acid) (PLA) was obtained from Ultimaker. The high expression reporter plasmid gWiz-GFP was purchased from Aldevron. The DNA (codon-optimized for human expression system, Genscript, #MC0101081) of SARS-CoV-2 surface glycoprotein without transmembrane domain was cloned into pCAGGS vector with in-fusion cloning technology (Takar #638916, Takara Bio USA).

### Design of Piezoelectric Pulse Generator and Microneedle Electrode Array.

The ePatch comprised a piezoelectric pulse generator and an MEA. The electric pulses were generated by a device derived from a common household piezoelectric stove lighter (Fig. 1*B*) (12). Briefly, a cylindrical chamber was 3D printed for housing a piezoelectric crystal harvested from a commercial lighter. The chamber had a wire connected to the piezoelectric crystal; the wire exited the chamber through its base. A hand toggle was attached at the top to provide the equivalent force utilized in a conventional lighter when it is pressed downward. The holder was 3D printed with PLA by a 3D printer (Ultimaker3, Ultimaker) (Fig. 1*C*).

The MEA was fabricated by assembling six rows of solid metal microneedles (Tech Etch) in an insulative holder. Each row had nine microneedles, each spaced 0.79 mm apart measured tip to tip. Microneedles with opposite electrical polarity were positioned adjacent to each other at a distance of 0.90 mm between rows. The pulse voltage and current profiles from the ePatch were measured by an oscilloscope (Tektronix TDS2014B Digital Storage Oscilloscope, Tektronix, Inc.) according to the electric circuit shown in *SI Appendix*, Fig. S1 *A* and *B*. The current through the skin during electroporation was calculated as the voltage across a 100-Ω resistor in series with the skin divided by the resistance of the resistor.

### Numerical Simulation of Electric Fields for Electroporation.

The electric field strength distribution was analyzed by numerical modeling using commercially available modeling software (CST Studio Suite 2019, Dassault Systèmes). The parameters for the numerical simulation of the electric field in the skin are shown in *SI Appendix*, Fig. S2. To simplify the model, we did not consider the conductivity changes of the permeabilized tissues during electroporation, thereby capturing the peak electric field strengths at the beginning of a pulse applied to previously untreated skin. The electric field simulation was done in electrostatic mode, where the rows of metal needle electrodes were set to static high and low potentials alternatively such that the voltage between the adjacent rows met the target voltage value. The medium between the needle tip sections was set using skin parameters to mimic the scenario when the microneedles penetrate the skin.

### Discrimination of Nonviable Cells and Electroporated Cells via Confocal Microscopy.

To study the effect of electroporation on cell viability and cell permeability in the skin, a cell-impermeable probe, SYTOX Green, was used to identify uptake by the transient cell membrane permeability caused by electroporation. SYTOX Green was coated on the microneedles before electroporation and used as an indicator for the transient permeability caused by electroporation. Another cell-impermeable probe, EB, was used as an indicator of nonviable cells caused by electroporation (65). BALB/c mouse skin was used as the tissue model.

Under anesthesia, the dorsal dermal hair was removed with a shaver, and then depilatory cream (Nair, Church & Dwight) was applied for 3 min. The skin was cleaned with wet gauze to remove the depilatory cream. Two days after hair removal, the mice were anesthetized with isoflurane (as described below), an MEA was pressed into the skin, and 5 or 20 piezoelectric pulses were applied. The mice were killed with carbon dioxide 10 min after electroporating the skin. The skin was harvested and submerged in phosphate-buffered saline (PBS) containing EB (50 μg/mL), incubated at 4 °C, and shaken for 1.5 h. The skin was washed three times with fresh PBS and imaged using a laser scanning confocal microscope (710 NLO, 20× objective, Carl Zeiss). SYTOX Green and EB were sequentially excited using an argon laser at 488 and 514 nm, respectively. Under the confocal microscope, the nonviable cells had red fluorescence from EB, and the electroporated cells had green fluorescence from SYTOX Green.

### Live Imaging of GFP Expression and Histological Examination of the Skin.

Rats were prepared under anesthesia 1 d before DNA delivery studies, by removing hair on their dorsal skin using a clipper, after which depilatory cream (Nair, Church & Dwight) was applied for 4 min and wiped clean with water. The animals were anesthetized in an induction chamber charged with 5% isoflurane in O_2_ by isoflurane vaporizer (SurgiVet Model 100, Smiths Medical), and then fitted with a standard rodent mask and kept under general anesthesia by setting the vaporizer at 1 to 2% isoflurane flow during the procedures.

Twenty microliters of PBS containing GFP plasmid (2.5 μg/μL) was injected ID to form a visible bleb in the skin. Electroporation pulses were applied to the injection site either with MEA or clamp electrodes 1 min after injection of the DNA. A specified number of microsecond pulses (1, 5, 10, or 20 pulses) were generated by ePatch to investigate the effect of pulse numbers on gene expression. A conventional benchtop electroporator (BTX Electro Cell Manipulator 600, Harvard Apparatus) with programmable pulse voltages was also used to study the effect of voltages of millisecond pulses on gene expression. The fluorescence intensity of GFP in the skin was monitored by an IVIS Spectrum CT in vivo imaging system (Perkin-Elmer) with region of interest tools on different days.

For histological examination studies, mouse skin in vivo was electroporated with 20 pulses by ePatch, and imaged under a stereomicroscope (Leica M80, Leica Biosystems) immediately after electroporation and again 3 h later. For hematoxylin/eosin (H&E) staining, the tissue was fixed overnight in 10% formalin buffer, then dehydrated by an automatic tissue dehydration system. The dehydrated tissue was embedded in paraffin, sectioned at 5-μm thickness by rotary microtome, and stained by Leica Autostainer XL (Leica Biosystems). The tissue was imaged by an inverted microscope (IX73, Olympus Life Science). For the fluorescence imaging of skin sections, a rat model was used. After the skin was harvested 12 h after electroporation, the tissue was embedded in Tissue-Plus O.C.T. Compound (Thermo Fisher Scientific) and frozen at −20 °C overnight before sectioning at 20-μm thickness using a freezing microtome (CryoStar NX70, Thermo Fisher Scientific). Tissue sections were imaged by laser scanning confocal microscopy (LSM 700, Carl Zeiss).

### Immunization Study in Mice.

For the mouse immunization study, we confirmed that the same electroporation parameters used in rats similarly produced strong GFP expression in mouse skin (*SI Appendix*, Fig. S9). BALB/c mice were randomized into five groups (*n* = 5 mice per group) that received injection of 10 μL of solution containing SARS-CoV-2 spike protein DNA vaccine in PBS: 1) 10 μg of DNA vaccine by IM injection, 2) 100 μg of DNA vaccine by IM injection, 3) 10 μg of DNA vaccine by ID injection, 4) 10 μg of DNA vaccine by ID injection followed by 20 pulses by the ePatch, and 5) PBS by ID injection as a negative control. The mice were anesthetized during the procedures by isoflurane, and the skin was wiped dry before ePatch application to avoid creating a conductive pathway outside the skin. Each animal received a second dose after 4 wk via the same procedures as the first dose. At week 7, blood was withdrawn by orbital sinus puncture, and the serum was separated.

### ELISA for SARS-CoV-2 Spike Protein Antibody Analysis.

ELISA was used to measure the titer of IgG against the spike surface protein of SARS-CoV-2 in the mouse serum. ELISA plates were coated with purified spike protein, then blocked with 5% bovine serum albumin. Serum samples were diluted 100-, 300-, 900-, 2,700-, 8,100-, and 24,300-fold with PBS containing 0.1% Tween 20 (PBST), then added separately to the ELISA plates and incubated at room temperature (20 °C to 25 °C) for 1 h, followed by washing three times with PBST. Horseradish peroxidase-conjugated goat anti-mouse antibodies were added and incubated for 1 h. The plates were washed again followed by the addition of 3,3′,5,5′-tetramethylbenzidine substrate to develop color. The reaction was terminated by a commercial stop solution (Thermo Fisher Scientific). The absorbance value was read at 450 nm by an ELISA plate reader (iMark Microplate Absorbance Reader, Bio-Rad). Optical density values at 450 nm (OD 450) were recorded and used as relative antibody expression levels in mice.

### Pseudovirus Neutralization Assay.

The SARS-CoV-2 spike protein pseudotyped virus was used in the neutralization assay. The pseudoviruses were produced by cotransfection of 293T cells with an env-deficient HIV-1 backbone plasmid DNA, pNL4-3.Luc.R-E-, and a DNA plasmid expressing the full-length SARS-CoV-2 spike protein flowing established protocols (66). The pseudoviruses were produced and self-packaged in 293T cells. The pseudovirus that was secreted into the supernatant of 293T cells was collected.

For analysis of serum neutralizing activities, 293T cells expressing angiotensin-converting enzyme 2 (ACE2) were seeded in a 96-well plate and grown overnight. Mouse serum samples were diluted 100-, 300-, and 900-fold with Dulbecco's Modified Eagle Medium (DMEM, Thermo Fisher Scientific). Each diluted sample (50 μL) was mixed with an equal volume of virus suspension (50 μL), followed by incubation at 37 °C for 1 h. Then, the samples containing the serum−pseudovirus mixture were added in triplicate to the wells of the 96-well plate seeded with ACE2-expressing 293T cells that were grown to 50% confluency. Six hours after infection, the suspensions were centrifugated at 1500 × *g* for 5 min, and the supernatant was removed and replaced with DMEM containing 5% fetal calf serum. After 48 h, the cells in each well were lysed, and the luciferase activity was determined as described previously (66). The neutralizing activity of immune sera was determined by the formula [(pseudovirus alone) − (pseudovirus+sera)]/[pseudovirus alone] × 100%.

### Safety Assessment.

Injection site reactions, including erythema and swelling, were assessed on the day of DNA vaccination, day 2 postvaccination, and day 7 postvaccination. Systemic adverse events were monitored throughout the study by veterinary staff.

### Statistical Analysis.

All data presented in this study represent mean ± SD. Statistical analysis was performed using a two-sided Student’s *t* test or ANOVA, with the software GraphPad Prism 8.0 (GraphPad). A value of *P* < 0.05 was considered significant.

## Data Availability

All data needed to evaluate the conclusion in the paper are present in the paper or *SI Appendix*.
